# The *Malaysian Journal of Medical Sciences*’ Performance Report for 2019

**DOI:** 10.21315/mjms2020.27.5.1

**Published:** 2020-10-27

**Authors:** Nour Azimah Zulkapli, Jafri Malin Abdullah

**Affiliations:** 1 Malaysian Journal of Medical Sciences, Universiti Sains Malaysia, Pulau Pinang, Malaysia; 2 Malaysian Journal of Medical Sciences, Universiti Sains Malaysia, Kubang Kerian, Kelantan, Malaysia

**Keywords:** journal performance, submission trend, impact factor

## Abstract

Once a year, we report the *Malaysian Journal of Medical Sciences* (MJMS)’ performance as a journal, along with important changes in the administration and achievements of the journal itself. We report here its submission trends, rejection rates, manuscripts accepted based on submitting country/region and impact factor scores for calendar year 2019.

## Introduction

On average, the *Malaysian Journal of Medical Sciences* (MJMS) continues to receive increased number of new manuscripts submission every year. In 2019, there were 453 new manuscripts submitted to MJMS (a 26% increase over that of 2018).

## Submission Trends

**Figure 1:**
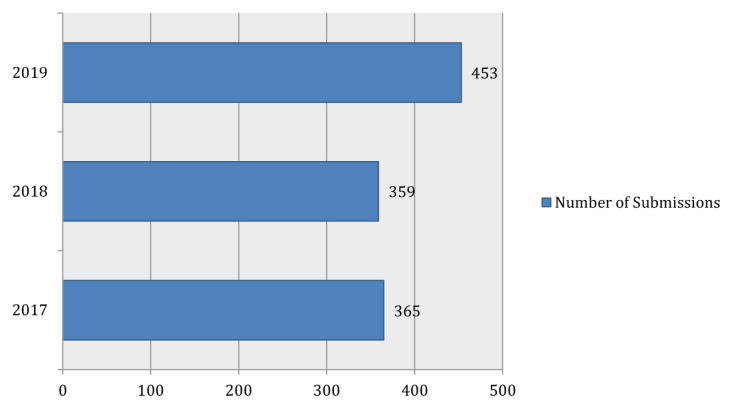
The number of new manuscript submissions via ScholarOne Manuscript^™^ system Source: https://mc.manuscriptcentral.com/maljms

**Figure 2:**
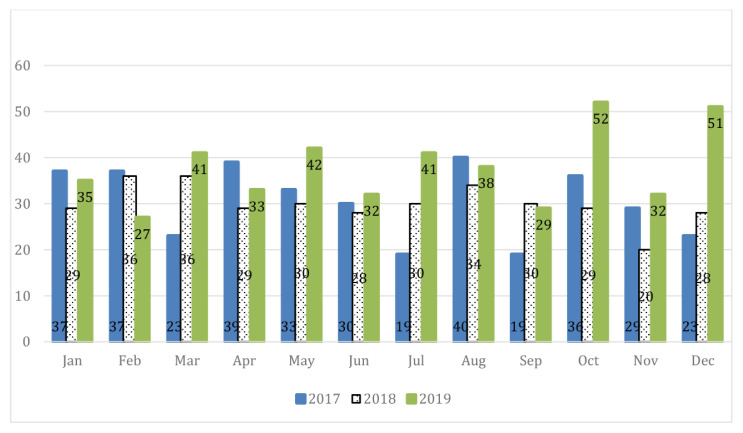
New manuscript submissions received over time Source: https://mc.manuscriptcentral.com/maljms

Figure 1 shows a brief picture of submission trends in three consecutive years although there was a slight decrease of total submission in the year 2018 compared to year 2017. Figure 2 shows the actual submission trends for every month of each concerned year. Obviously, the increment of new manuscript submission at almost two-fold compared to year 2018 started in October 2019 and happened again in December 2019.

## Rejection Rates

New manuscripts submitted to MJMS would initially be assessed to a lesser degree by the Editorial Office to ensure they conform to MJMS requirements and the subject discussed was relevant to MJMS’ main focus. At this level, only a few numbers were desk-rejected and did not make it to the peer-review process. A considerable number of rejections happened to manuscripts which had succeeded to undergo at least the first hurdle of peer-review process and had gone through intense judgement in terms of scientific merit, not technical mistakes. In another word, it depended more on the quality of the manuscripts, not the number of reviewers (1).

Data derived from the ScholarOne Manuscripts^™^ system (Table 1) showed the rejection rate of manuscripts based on early screening and final decision in 2017–2019. The rejection rate increased to 83.1% in 2019 compared to the rate in two previous years.

**Figure 3:**
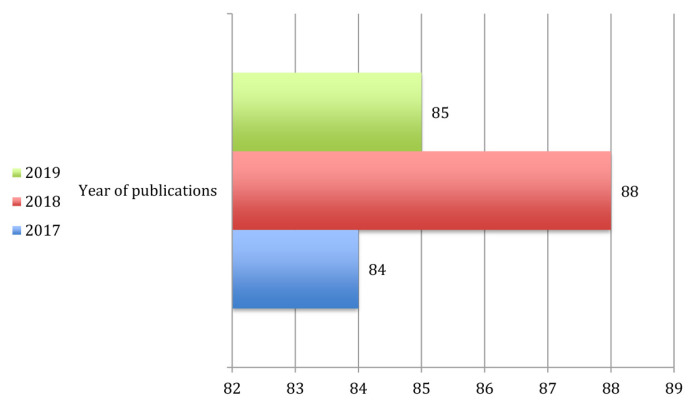
The number of manuscript published Source: https://mc.manuscriptcentral.com/maljms

However, the highest rejection rate in 2019 did not have any relation to the number of published articles although there was a slight decrease in this particular year compared to year 2018 (Figure 3). This was because MJMS always had a complete number of accepted articles to be published in an issue.

## Manuscripts Accepted Based on Submitting Country/Region

Table 2 shortlisted a number of developing countries/regions that had contributed to the highest number of manuscripts in each respective year. As expected, Malaysia had been the highest manuscript contributor and at the same time, was the country with highest number of accepted manuscripts followed by Iran, Indonesia and India.

As seen in the table concerned, most authors of each countries/regions had made huge manuscript contributions to MJMS but unfortunately, only about one-third of the manuscripts were accepted for publication after peer-reviewed.

It was assumed that among the main reasons of the rejections that led to only a small number of acceptances were:

- the study was too basic
- the study did not follow proper scientific methods, and had poor statistical methodologies and analyses
- arguments were illogical or invalid
- methods were incomplete
- the study were poorly presented and the language used were inconsistent

## Impact factors

Impact factors, as one citation measure, were useful in establishing the influence journals had within the literature of a discipline (2). The impact factor (IF) 2018 of Malaysian Journal of Medical Sciences was 0.92, which was computed in 2019 as per it’s definition. MJMS’s IF was increased by a factor of 0.11 and the approximate percentage change is 13.58% when compared to the preceding year 2017, which showed a rising trend (3).

## Achievement

MJMS had received the CRÈME Award 2019 (the Best Malaysian Journal) for the second time, after the first award as the Potential Journal received in 2016 from the Ministry of Higher Education, Malaysia.

## Figures and Tables

**Figure 4:**
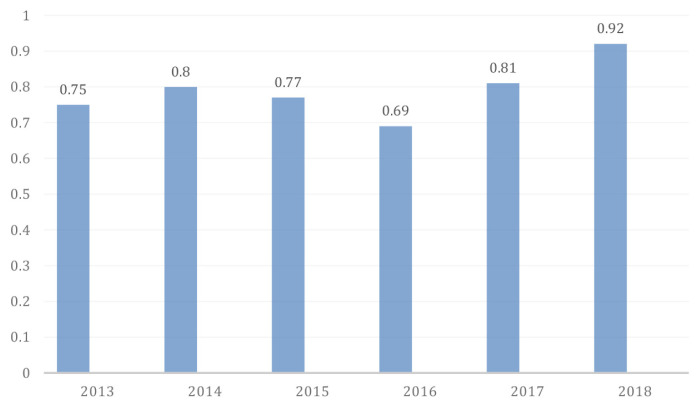
Impact factor of MJMS (4)

**Table 1:** Rejection rates based on early screening and manuscript decision

Year	2017	2018	2019
Rejection rate (%)	77.3	78.4	83.1

Source: https://mc.manuscriptcentral.com/maljms

**Table 2:** The countries/regions with total manuscript submissions and accepted manuscripts in respective years

Country/Region	2017		2018		2019	
	Accepted (*n*)	Submission (*n*)	Accepted (*n*)	Submission (*n*)	Accepted (*n*)	Submission (*n*)
Malaysia	42	131	59	148	40	144
Iran	5	45	12	83	9	51
Indonesia	5	24	2	19	8	37
India	3	45	3	38	1	29
Nigeria	0	17	5	23	1	21
Saudi Arabia	2	7	0	8	1	13
Pakistan	0	4	0	7	0	8
Thailand	3	4	1	3	3	7
Turkey	0	2	0	6	0	4

Source: https://mc.manuscriptcentral.com/maljms
